# Dr. Wiley and the “Interests”

**Published:** 1911-08

**Authors:** 


### EDITORIAL DEPARTMENT.

I ask the indulgence of my readers if they should look for "able editorials" in this issue. My health is very much impaired in consequence of an attack of "grippe" in March and to escape the heat of Austin and to take my first real vacation in twenty-five years—an "outing"—I am camped on the banks of the beautiful, clear, ice cold San Marcos river, and, like Anteus of old, I am abstracting strength from contact with Mother Earth. I catch a black bass occasionally, but ordinarily do nothing but eat, sleep and just "laze" or lie in a hammock in the shade and read—read—anything funny I can get hold of.

---

Dr. Wiley and the "Interests."—"He can and he can't, he will and he won't, he'll be damned if he does and damned if he don't." It is remarkable that if a man does his duty well and conscientiously—which he is sworn to do—there are those who blame him and seek to put him out. This is the case with Dr. Wiley. He has made the substitutors and adulterators squeal, and they are after his scalp. "Nothing ever felt the halter draw and entertained a good opinion of the law."

